# A Novel Multilayered RFID Tagged Cargo Integrity Assurance Scheme

**DOI:** 10.3390/s151027087

**Published:** 2015-10-23

**Authors:** Ming Hour Yang, Jia Ning Luo, Shao Yong Lu

**Affiliations:** 1 Chung Yuan Christian University, Chung Pei Road, Chung Li District, Taoyuan 32023, Taiwan; E-Mail: lumosac@gmail.com; 2 Ming Chuan University, Deming Road, Guishan District, Taoyuan 33348, Taiwan, E-Mail: deer@mail.mcu.edu.tw

**Keywords:** radio-frequency identification, multilayered grouping proof, supply chain management, anonymity, multi-session attacks, concurrency attacks

## Abstract

To minimize cargo theft during transport, mobile radio frequency identification (RFID) grouping proof methods are generally employed to ensure the integrity of entire cargo loads. However, conventional grouping proofs cannot simultaneously generate grouping proofs for a specific group of RFID tags. The most serious problem of these methods is that nonexistent tags are included in the grouping proofs because of the considerable amount of time it takes to scan a high number of tags. Thus, applying grouping proof methods in the current logistics industry is difficult. To solve this problem, this paper proposes a method for generating multilayered offline grouping proofs. The proposed method provides tag anonymity; moreover, resolving disputes between recipients and transporters over the integrity of cargo deliveries can be expedited by generating grouping proofs and automatically authenticating the consistency between the receipt proof and pick proof. The proposed method can also protect against replay attacks, multi-session attacks, and concurrency attacks. Finally, experimental results verify that, compared with other methods for generating grouping proofs, the proposed method can efficiently generate offline grouping proofs involving several parties in a supply chain using mobile RFID.

## 1. Introduction

Mobile radio frequency identification (RFID), a widely adopted technique in supply chain management (SCM), involves using a mobile reader to scan RFID tags [1,2]. In each SCM stage, RFID tags can be employed to store cargo information and facilitate autonomous stocktaking, thereby improving inventory management and accelerating the retail cycle [3]. RFID tags can also be employed to track and locate cargo, which not only improves order management [3], but also reduces management costs. For example, by adopting an RIFD SCM system, Wal-Mart reduced its annual distribution costs by approximately 6%–7%, an equivalent saving of US$1.4 billion [4]. 

Although tracking cargo through RFID is easier than using other logistics management methods, malicious members of a supply chain can steal cargo during the transfer process [5,6]; consequently, delivery of all cargo cannot be guaranteed. Annually, cargo theft accounts for approximately US$30 billion in losses worldwide [4]. However, when determining whether the suppliers, transporters, and recipients are suspects, disputes may arise when the actual suspect cannot be confirmed [7]. To solve this problem, Zhou and Rodrigues have proposed a smart grid based infrastructure to verify the generated codes for each recipient [8]. Lo *et al.* [9] proposed a cargo tracking system utilizing RFID. The tracking system provides the ability to report the real-time locations of the cargo and its flow. RFID systems have been designed to generate grouping proofs for all RFID tags during cargo transfer [10,11,12]. Accordingly, in the event of a dispute, transporters can use the receipt proofs as evidence of having delivered the cargo. Moreover, by generating a receipt proof when the cargo is delivered, the recipient provides undeniable proof that they have received their shipment in full.

However, if a poorly designed integrity check mechanism is used, malicious users can exploit the mechanism, thereby compromising its credibility. For example, attackers can record the messages communicated between tags and readers and then replay them to generate bogus proofs that can pass authentication protocols, despite nonexistent tags being used in the attack. Saito and Sakurai [13] employed timestamps generated by a verifier to replace random numbers [14] to protect against replay attacks. However, tag impersonation attacks can be used to exploit generating timestamps in systems that generate multiple grouping proofs simultaneously. Therefore, Peris-Lopez *et al.* [15,16] have proposed a protocol for generating grouping proofs that protect against tag impersonation attacks and safeguards the method proposed by Yu, Hou, and Chiang [17] by not only protecting against multisession attacks, in which malicious users intercept pieces of grouping proofs transmitted in the same time zone and then generate a composite bogus proof, but also protecting RFID systems from most attacks targeting online grouping proofs. However, because generating the grouping proof protocol requires writing data to an RFID tag multiple times, concurrency attacks can occur when multiple readers concurrently generate grouping proofs for the same RFID tag, causing messages from the readers to interactively overwrite the tag content generated by each reader in the tag, thus generating the erroneous grouping proofs. Therefore, Lin, Lai, Tygar, Yang, and Chiang [18] proposed a protocol that instantly generates grouping proofs to protect against concurrency attacks that prevent grouping proofs from being generated. 

When transporters receive or deliver cargo in areas where mobile readers cannot connect to the verification server (verifier) for authentication, the server cannot generate a trustworthy time value to initiate generating the grouping proofs [10]. Therefore, Sun, Ting, and Chang [19] and Hermans and Peeters [20] have utilized the timeout mechanism of RFID tags to ensure that the grouping proofs are accurate. When group members cannot complete the protocol because they are not within range of a mobile reader, the reader cannot obtain the grouping proofs or ensure that all of the RFID tags are in the same interval when the proofs are generated. Lo and Yeh [21] and Ma, Lin, Wang, and Shang [22] have employed additional active tags or trusted devices that generate timestamps to ensure that the time difference between timestamps is within a time threshold before responding to a mobile reader’s requests for the generated proofs; thus, all the of the RFID tags involved in the grouping proofs must be present at the same interval. 

In addition to the correctness of the generated grouping proofs, the conventional grouping proofs [14,23,24,25,26,27,28] are generated by all tags on site one after another, thus making these tags incapable of simultaneously computing the pieces of proof assigned to the tags. The calculation time for generating this type of grouping proof increases with the number of tags; thus, it is unsuitable for cargo with a considerable number of RFID tags, which is common in most supply chains. Lien, Leng, Mayes, and Chiu [29] proposed a method that satisfies the exclusiveness of commutative property or is capable of combining the pieces of proof computed by the tags through XOR operands in order to generate grouping proofs. The method enabled the reader to request that proofs be generated by other tags before the pieces of proof have been reconstructed. Without needing to consider the sequence of requests, the request order can be directly combined to generate the final grouping proof. In addition, parallel computing accelerates the generation of grouping proofs. Jantarapatin, Mitrpant, Tantibundhit, Nuamcherm, and Kovintavewat [30] and Nuamcherm, Kovintavewat, Tantibundhit, Ketprom, and Mitrpant [31] have improved the security of message requests for grouping proofs by enabling mobile readers to directly broadcast the random number incorporated in the tags. All tags must simultaneously use both the key shared by the verification server and encrypt to obtain the random number sent by the reader in order to obtain protocol for generating the pieces of proof. Similarly, the final grouping proof is produced by reconstructing the pieces of proof generated by each tag through a process of exclusion or combination, which reduces the amount of time taken for generating the proof. However, with this method, compromised readers can be exploited to insert random numbers in the request messages before transmitting them to the tags; after recovering the pieces of proof from the tags, a piece of proof with a tag that is the same as the tag of the inserted random number can be used to generate bogus grouping proofs that can pass authentication, despite not having the tags. Thus, Sun, Ting, and Chang [19] proposed using message broadcast to render it impossible for readers to generate a bogus grouping proof with nonexistent tags through encrypting the returned messages of all the participating tags using a shared key. Yen, Lo, and Wu [32] indicated that when a reader is online (*i.e*., connected to a network), all tags should first be authenticated, and the grouping proofs should then be generated and transmitted to a backend verifier; thus, completion can be achieved by broadcasting only two messages. Hermans and Peeters [20] employed an additional timestamp to initiate the procedure of generating grouping proofs and signing the final collected grouping proof, thus achieving the same outcome except in an offline environment.

The time taken for generating pieces of proof can be reduced considerably by using parallel computing because the requests for grouping proofs are broadcast to all RFID tags. However, when these tags transmit the pieces of proof to the reader, collisions occur because of simultaneously receiving a high number of tag messages. If messages are sent using anticollision mechanisms, such as tree-walking or the aloha protocol of ISO-18000 [33], then the time taken for responding to messages increases [34,35]; thus, malicious users can exploit this time difference to generate a legitimate grouping proof from a group of tags that are not in the same time interval [36]. Therefore, Leng, Lien, Mayes, and Markantonakis [37] proposed a subgrouping grouping proof that minimized the number of collisions by using fewer grouping tags. This method involves dividing groups of tags into several subgroups and generating proofs sequentially; however, because only one reader is employed to generate the grouping proofs, retransmitting messages across subgroups can only be performed in the specific sequence; thus employing parallel retransmission does not improve the effectiveness. Moreover, the scanning restrictions on the maximum number of tags that can be read by the reader [38,39] render this method unfeasible in logistics management when a high number of tags requiring scanning are involved.

Therefore, the present study proposed a method for generating multilayered grouping proofs to resolve the problem of needing to scan generated grouping proofs in batches because of the limited number of tags that can be scanned using a reader [38,39], which, in a supply chain environment, would invalidate the grouping proof because the processing time would exceed its time threshold. The goal of our method is proposing a hierarchical management framework that enables several readers to simultaneously generate pieces of proof; subsequently, the final grouping proofs are generated by an authorized reader. In addition, regardless of whether the reader can connect to the backend verifier, the cargo transporter can use the reader to collect tag information to generate proofs. When the messages are transmitted in an online environment, the verifier can confirm whether the tags were generated by readers in the same location. Thus, the proposed method is effective for (1) generating grouping proofs for a supply chain in which a considerable number of tags must be scanned simultaneously; (2) reducing the time required for generating proof concurrently through multiple readers when a high number of tags are involved; (3) providing undeniable proof of delivery for recipients, suppliers, and transporters by generating proofs before every cargo transfer and by acquiring signatures from all relevant personnel as evidence that the cargo was received in full and by the intended recipient; (4) ensuring that the tags are secure from replay, eavesdropping, and impersonation attacks, and as well as most attacks designed to generate bogus grouping proofs; (5) ensuring data confidentiality through using anonymous tags; (6) ensuring that the supply chain’s cargo routes remain confidential; (7) automatically determining whether the transporters deliver the cargo on time and according to order; and (8) dynamically scaling the number of cargo tags according to the scan rate at which the mobile reader scans to generate grouping proofs. 

The remainder of this paper is organized as follows. Section 2 introduces the environmental hypothesis for the grouping-proof protocol applied to many RFID readers, as well as the relationships among the tags, readers, and certain members in a supply chain. In addition, the initialization process and methods for generating and authenticating grouping proofs are explained in detail. Subsequently, Section 3 investigates the security of the proposed protocol and compares it with that of other grouping proofs. In Section 4, the computation effectiveness of the proposed method is analyzed and compared with that reported by related studies. Finally, Section 5 offers the conclusions for this study.

## 2. Cargo Inspection Management of Mobile Logistics

**Figure 1:**
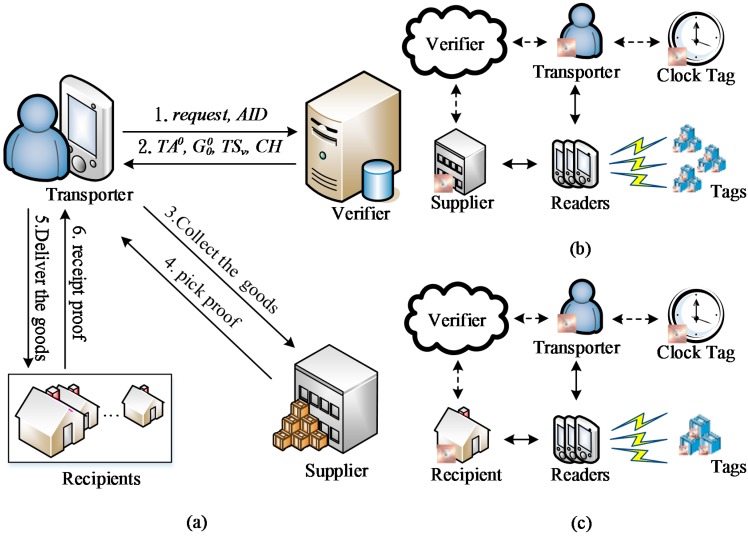
Process of supply chain distribution and grouping proof: (**a**) steps of the supply chain distribution; (**b**) generate the grouping proofs for pick up and delivery; (**c**) generate the grouping proofs for cargo delivery.

The method proposed in this study can be applied to improve SCM. As depicted in Figure 1a, the proposed method can be adopted to automatically generate grouping proofs to facilitate using mobile RFID readers to manage cargo. To automate the scanning process for transporters delivering cargo, suppliers, transporters, and recipients adopting the protocol must first register their mobile RFID devices on a verifier (*i.e*., a server for backend authentication). In addition, the threat model developed is based on the hypothesis that malicious users can conduct eavesdropping attacks by intercepting the messages transmitted by RFID readers and tags. In addition, transporters might exploit the proposed protocol through the following two malicious behaviors: (1) providing a bogus proof to conceal stolen cargo; and (2) tampering with the proof timestamp to conceal delayed deliveries caused by negligence. Therefore, in this study, a clock tag was incorporated into the transporter’s reader to ensure that the timestamp generated under offline conditions is trustworthy for when the reader cannot connect to the verifier.

As indicated by Step 1 in Figure 1a, when the transporter requests the verifier to authenticate the delivery of cargo to *n* recipients, denoted as $P^{1},\;P^{2},\;\ldots ,P^{n}$, the delivery of cargo to any recipient can be expressed as shown in Equation (1):

(1) $$\;T_{\;}^{q}=\left\{TID_{i}^{q}│\;\forall i\;TID_{i}^{q}\in P_{\;}^{q},1\leq i\leq \delta q,\delta q\in {\mathbb{Z}}^{+}\right\}$$

where $TID_{1}^{q}$,$TID_{2}^{q}$,…,$TID_{\delta q}^{q}$ denote the RFID tag codes; *δq* represents the number of RFID tags; and $PID_{\;}^{q}$ is the recipient’s RFID code, which is incorporated into the tags.

Assume that a supplier must deliver cargo with a group of RFID tags, denoted as $T^{0}=\;T^{1}\cup^{\text{​}}\;T^{2}\cup^{\text{​}}\ldots \cup^{\text{​}}\;T^{n}$, where *n* denotes the number of recipients. To ensure that the transporter can immediately check the integrity of cargo delivered to recipient $P_{\;}^{q}$, the verifier applies Equation (2) to obtain a verification code indicating the integrity of the delivered cargo:

(2) $$TA_{\;}^{q}=TH_{1}^{q}||TH_{2}^{q}\left|\left|\ldots \right|\right|TH_{\delta q}^{q},where\;\forall i\;TH_{i}^{q}=H\left(TID_{i}^{q}\left|\left|Kt_{i}^{q}\right|\right|TS_{v}^{\;}\right)$$

where $TA_{\;}^{q}$ is the verification code; $Kt_{i}^{q}$ denotes the shared key for the verifier and the tag $TID_{i}^{q}$; and $TS_{v}^{\;}$ is a timestamp generated by the verifier.

To enable the RFID reader to encrypt the multicast messages transmitted to the cargo tags and to establish a secure multicast channel between the readers and tags [40], the verifier generates the group keys for the *k*-ary tree with $GK_{0}^{q}$ as the starting node (a detailed explanation of key tree is provided in Section 2.1). At Step 2 in Figure 1a, the verifier transmits the following data to the transporter’s reader: a group of verification codes $TA^{0}=\left\{TA^{1},TA^{2},\;\ldots ,TA^{n}\right\}$ for *n* recipients; a key tree comprising the group of tags $GK_{0}^{0}=\left\{GK_{0}^{1},GK_{0}^{2},\ldots ,GK_{0}^{n}\right\}$ from the recipients; the timestamp $TS_{v}^{\;}$; and verification codes for the clock tags generated in the offline phase. Figure 1b shows that the reader generates verification codes for the transporter, supplier, all of the RFID tags involved in delivering the cargo, and a receipt proof for the cargo delivery. Subsequently, the check codes generated by the transporter’s reader and the verifier are compared to confirm whether they are identical in order to ensure the integrity of the cargo and that it has been received in full.

As indicated in Figure 1c, when the transporter delivers cargo to each recipient, the cargo tags and recipient’s RFID tag are scanned using the reader, which then generates receipt proofs and verification codes for each recipient. The integrity and correctness of the delivered cargo are confirmed by ensuring that the verification code issued by the verifier and the receipt proof and verification code generated by the transporter are consistent. The reader transmits the pick proofs and receipt proofs to the verifier as soon as a connection is established. In the event of a dispute (e.g., the recipient denying having received the cargo), the receipt proof is evidence that the transporter has already delivered the cargo in full, as illustrated in Step 6 of Figure 1a. By contrast, if the transporter denies that the cargo has been consigned by the supplier, then the pick proof is evidence that the transporter has already collected the cargo, as indicated in Step 4 of Figure 1a. Moreover, if the recipient notices that the cargo content differs from the consignment note, then the backend verifier can cross-reference the pick proof and receipt proof. Should the tags for the receipt proofs and pick proofs be identical, then the error is associated with the quantity shipped by the supplier. A discrepancy between the receipt proof and pick proof indicates that the transporters has lost cargo in route. Thus, the grouping proofs solve and clarify problems regarding lost cargo.

However, some problems remain unresolved in this method of logistics verification; when the reader cannot connect to the verifier, trusted proofs cannot be generated securely. Regarding the method proposed in this study, identical procedures are used for picking up cargo from suppliers and transporting cargo to recipients. Although pick proofs must be generated from cargo tags when suppliers consign cargo for deliver to multiple recipients, the method proposed in this study is no different except for the number of tags, the personnel engaged in the process, and the identification code of the reader. Without loss of generality, the subsequent sections discuss the following three phases for generating grouping proofs, which is achieved through the transporter and recipient using the identification codes AID and $PID_{\;}^{q}$, respectively: (1) an initialization phase for generating the group key tree; (2) an integrity verification phase for generating grouping proofs and integrity check codes for when the multilayered reader is used in offline phase; and (3) a dispute resolution phase, in which all of the grouping proofs are examined to verify the cargo delivery process in the event of a dispute.

### 2.1. Initialization Phase

**Figure 2:**
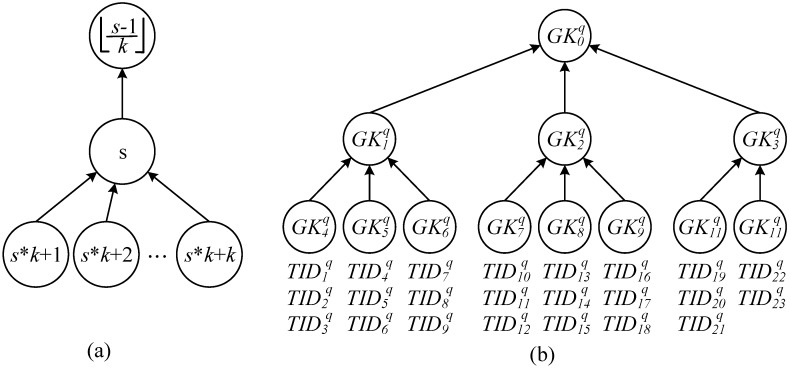
Group Key tree of cargo tags of recipient $P_{\;}^{q}$: (**a**) Rule for numbering among group keys; (**b**) key tree for 3-ary group tags.

When a cargo shipment with a tag collection $\;T_{\;}^{q}$ is delivered to recipient $P_{\;}^{q}$, the reader requests the verifier to establish a secure multicast connection to ensure that the generated grouping proofs accord with those on the recipient’s reader and that messages can be transmitted to the *δq* tags in $\;T_{\;}^{q}$. Therefore, the verifier generates a *k*-ary group key tree with a height difference of the subtree of ≤1 (tree height of $h_{max}=\lceil \log_{k}\left(\delta q/k\right)\rceil$) for the shared key $Kt_{i}^{q}$ available to all tags $TID_{i}^{q}$. In summary, the group keys that can transmit messages to *δq* tags in $\;T_{\;}^{q}$ are defined as $GK_{0}^{q}$, as illustrated in Figure 2. Figure 2a shows the numbering sequence that is generated when the group number of a certain node in the *k*-ary group key tree is $G_{s}^{q}$: from top to bottom, from left to right, the number of parent nodes is $G_{\left\lfloor (s-1\right)/k\rfloor }^{q}$, and the number of the child nodes ranges from $G_{s*k+1}^{q}$ to $G_{s*k+k}^{q}$. Figure 2b indicates an example of a 3-ary key tree generated by $\;T_{\;}^{q}$, a set of 23 tags; the group key $GK_{2}^{q}$ is employed to encrypt the multicast messages transmitted to the tags numbering from $TID_{10}^{q}$ to $TID_{18}^{q}$, and the tags $TID_{10}^{q}$, $TID_{11}^{q}$, and $TID_{12}^{q}$ decrypt the multicast messages encrypted with the group key $GK_{7}^{q}$ by using the keys $Kt_{10}^{q}$, $Kt_{11}^{q}$, and $Kt_{12}^{q}$ shared with the verifier.

Therefore, the nodes with the group number of $G_{s}^{q}$ are composed by 1 to a number of *k* subtrees, with Equation (3) indicating the group key incorporated by any node $G_{s}^{q}$. Group key $GK_{s}^{q}$ exists in all the parent groups numbered $G_{spar}^{q}$ that incorporate $G_{s}^{q}$ and satisfy the intersection of group number $G_{s}^{q}\;$and $G_{spar}^{q}$ equaling $G_{s}^{q}$. The difference set between $G_{0}^{q}$ and $G_{spar}^{q}$ and the intersection between $G_{0}^{q}$ and $G_{s}^{q}$ as an empty set is as indicted in Equation (4).

$$G_{s}^{q}=\left\{GK_{l}^{q}│\forall l\;GK_{l}^{q}\in G_{s}^{q},s*k^{h}+\frac{k^{h}-1}{k-1}\leq l\leq s*k^{h}+\frac{k\left(k^{h}-1\right)}{k-1},h\in {{\mathbb{Z}}_{0}}^{+},s\in {{\mathbb{Z}}_{0}}^{+}\right\}$$

(3) $$G_{spar}^{q}=\left\{GK_{s}^{q}\in G_{\left\lfloor \frac{s-\frac{k^{h}-1}{k-1}}{k^{h}}\right\rfloor }^{q},\;h\in {{\mathbb{Z}}_{0}}^{+},\;s\in {{\mathbb{Z}}_{0}}^{+}\right\},\;\;\;\forall s\;GK_{s}^{q},\text{where }G_{s}^{q}\cap^{\text{​}}G_{spar}^{q}=G_{s}^{q}$$

and

(4) $$\left(G_{0}^{q}-G_{spar}^{q}\right)\cap^{\text{​}}G_{s}^{q}=\emptyset$$

**Table 1:** Definition of symbols.

V	Verifier: a third-party verification server to reinspect grouping proofs
S	Supplier: shipping supplier
A	Transporter: transporter who delivers cargo
$P_{\;}^{q}$	Recipient: *q*-th recipient who receives the cargo
C	Clock Tag: a third-party clock tag providing time for the system in offline phase
AID	Identification code for A
$CID_{\;}^{\;}$	Identification code of a trusted and active third-party C
$RID_{0}^{\;}$	Identification code of the reader used by a
$PID_{\;}^{q}$	Identification code of $P_{\;}^{q}$
$RID_{j}^{q}$	Identification code of the j-th reader used by $P_{\;}^{q}$
$TID_{i}^{q}$	Identification code of the i-th tag for $P_{\;}^{q}$
$G_{s}^{q}$	s-th group code for $P_{\;}^{q}$
$TH_{i}^{q}$	Verification hash value for $TID_{i}^{q}$
Kc	Shared key for C and V
$Kr_{j}^{q}$	Shared key for $RID_{j}^{q}$ and V
$Kt_{i}^{q}$	Shared key for $TID_{i}^{q}$ and V
$GK_{s\;}^{q}$	Shared key for $G_{s}^{q}\;$and V
$SK_{j}^{q}$	Session key among readers
$PK^{a}$	Public key for a
$PR^{a}$	Private key for a
$PK_{\;}^{q}$	Public key for $P_{\;}^{q}$
$PR_{\;}^{q}$	Private key for $P_{\;}^{q}$
$Nr_{j}^{q}$	Random number generated by $RID_{j}^{q}$
$Nt_{i}^{q}$	Random number generated by $TID_{i}^{q}$
$Nc_{\;}^{\;}$	Random number generated by C
$Na_{\;}^{\;}$	Random number generated by a
$Np_{\;}^{q}$	Random number generated by $P_{\;}^{q}$
$TS_{v}^{\;}$	Timestamp generated by V
$TS_{c}^{\;}$	System time of C
ΔT	Time threshold for generating grouping proofs
E(key,Msg)	Encryption function generated by message (Msg) through an employment of symmetric key (key)
Sign(key,Msg)	Signing function generated by Msg through an employment of private key (key)
MAC(key,Msg)	Function for message authentication code generated by Msg by an employment of Key
H(Msg)	Message authentication code generated from an employment of hash function by Msg
$FO_{\;}^{q}$	Judgement of whether the grouping proof for $P_{\;}^{q}$ is generated in online or offline phase

In addition, this study defined the ⌈δq/k⌉-number of key trees coded from $\left\lceil \frac{\left(\delta q/k\right)-1}{k-1}\right\rceil$ to $\lceil \frac{\left(\delta q/k\right)-1}{k-1}\rceil +\lceil \delta q/k\rceil -1$, that directly connects with the nodes on the tags, as leaf group, as indicated in Equation (5) and represented by $G_{leaf,m}^{q}$. For example, tag codes $TID_{1}^{q}$, $TID_{2}^{q}$, and $TID_{3}^{q}$ connect with the leaf node $G_{leaf,1}^{q}$ of group code $G_{4}^{q}$.

(5) $$G_{leaf,m}^{q}=\left\{TID_{l}^{q}│\forall l\;TID_{l}^{q}\in G_{leaf,m}^{q}\;,\;\left(m-1\right)k+1\leq l\leq mk,1\leq m\leq \lceil \frac{\delta q}{k}\rceil \right\}$$

Table 1 provides the definition of the symbols used in the protocol.

### 2.2. Integrity Verification Phase: Grouping Proof Protocol of a Multilayered Reader

After the transporter delivers the cargo to the recipient and simultaneously generates grouping proofs using the reader with a maximum reading capacity of *r*, the group keys are distributed to several mobile readers from the transporters’ reader $RID_{0}$ to securely multicast messages to *δq* tags via the recipients’ readers, thus enabling each reader to receive the maximum number of tags by performing only one multicast; in other words, the grouping proof is generated using the minimum number of group keys. During the initialization phase, Equation (3) is employed to generate the group keys and construct a complete *k*-ary key tree for $RID_{0}$ with a tree height of h = $\left\lceil \log_{k}\left(\delta q/k)\right\rceil -\lfloor \log_{k}\left(r/k\right)\right\rfloor$ for multicasting $k^{\left\lfloor \log_{k}r\right\rfloor }$ tags; in other words, the key tree satisfies the maximum number of reading limits, *r*, and can encrypt the maximum number of tags with group keys, thereby forming a complete subtree. Therefore, when the number of tags that $k^{h}$ group key (whose height h equals that of the key tree) can encrypt $k^{h}\times k^{\left\lfloor \log_{k}r\right\rfloor }$ tags) equals *δq*, the complete key tree with successive group keys with a multicast range from 1 to r for multicasting $k^{\left\lfloor \log_{k}r\right\rfloor }$ tags is derived, which also satisfies the nonrepetition requirement for the tags.

(6) $$f\left(a\right)=\lfloor \frac{\lceil \frac{\left(\delta q/k\right)-1}{k-1}\rceil +\lceil \delta q/k\rceil -1-\frac{k^{a}-1}{k-1}}{k^{a}}\rfloor$$

**Figure 3:**
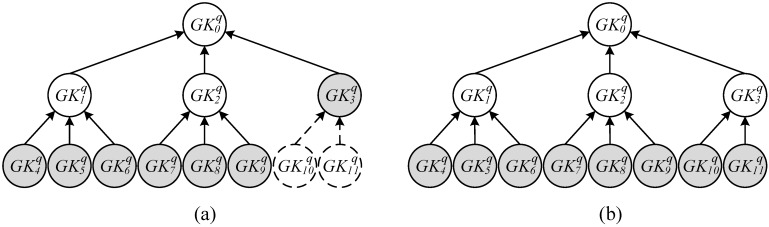
Examples showing group key selection in an incomplete group Tree: (**a**) the number of remaining tags RA≤  reading capacity of r tags; (**b**) RA > r.

However, if $k^{h}\times k^{\left\lfloor \log_{k}r\right\rfloor }$ > δq, then the *k*-ary key tree (Figure 3) would be incomplete. To enable the reader to scan all of the tags with the least number of group keys, the system must perform a search to determine whether any incomplete tree contains a group key that can encrypt a particular number of tags, the number ranging between $k^{\left\lfloor \log_{k}r\right\rfloor }$ and *r*. Because an incomplete subtree would appear in the subtree with the maximum number of leaf nodes, Equation (6) is applied to determine $f\left(\lfloor \log_{k}r\rfloor \right)$ in order to determine the parent node code at the level of $\left\lfloor \log_{k}r\right\rfloor$ (height = h−1) above the maximum leaf node code level $\lceil \frac{\left(\delta q/k\right)-1}{k-1}\rceil +\lceil \delta q/k\rceil -1$. Subsequently, Equation (3) is employed to determine all of the leaf group codes in the encrypted nodes; also in addition, Equation (5) is applied to compute RA, the total number of tags that can be read. As expressed in Equation (7), when the maximum number of *r* tags scanned by the reader can contain RA number of tags, a group key with the code $f\left(\lfloor \log_{k}r\rfloor \right)$, which satisfies the number of tags between $k^{\left\lfloor \log_{k}r\right\rfloor }$ and *r* quantity, is obtained. Consequently, the group key with the code $f\left(\lfloor \log_{k}r\rfloor \right)$ is selected as the first key $R_{f}$; else, the smallest code in which the message can multicast to $k^{\left\lfloor \log_{k}r\right\rfloor }$ number of tags can be selected as the first key $R_{f}$.

Subsequently, Equation (8) was employed to compute that under these two conditions, another group key code $R_{e}$ larger than $R_{f},$ enabling the key with codes ranging between $R_{f}$ and $R_{e}$ to be capable of encrypting all tags, with none of the two keys repeatedly encrypting the same tag.

(7) $$R_{f}=\{\begin{array}{c} f\left(\lfloor \log_{k}r\rfloor \right)\;,\;RA\leq r\\ f\left(\lfloor \log_{k}r\rfloor \right)+1,\;RA>r \end{array}$$

(8) $$R_{e}=\{\begin{array}{c} k*R_{f}\;,k*R_{f}<\lceil \frac{\lfloor \delta q/k\rfloor -k}{k-1}\rceil +\lceil \delta q/k\rceil -1\\ \lceil \frac{\lfloor \delta q/k\rfloor -1}{k-1}\rceil +\lceil \delta q/k\rceil -1,k*R_{f}\geq \lceil \frac{\lfloor \delta q/k\rfloor -k}{k-1}\rceil +\lceil \delta q/k\rceil -1 \end{array}$$

For example, Figure 3a is a distribution diagram of Figure 2b that involves the results obtained from the 23 group keys tagged on the *k* = 3-ary group key tree for the reader with a reading capacity of *r* = 6. According to $\left\lceil \log_{3}\left(23/3\right)\rceil -\lfloor \log_{3}\left(6/3\right)\right\rfloor$=2-0=2, the group key with the tree height of 2 can multicast message to three tags and encrypt the maximum number of tags within the capacity that can be read by the reader. In addition, of the parent node located in the level of $\lfloor \log_{3}6\rfloor =1$ above the group key $\;GK_{11}^{q}$ of the largest leaf group within an incomplete key tree coded 11, the leaf group keys $GK_{10}^{q}$ and $GK_{11}^{q}$ incorporated in key $GK_{3}^{q}$, with a tree height of 1, can read three and two tags, respectively. This study computed that an incomplete subtree with the height of 1 can read a total of 5 tags, which was less than 6, the number of tags that can be simultaneously read by a reader; in addition, the tags that can be encrypted by the largest leaf node was already incorporated within the group key $\;GK_{3}^{q}$ $k\times R_{f}=3\times f\left(2\right)=$9→9 <11). Therefore, a reader generates grouping proofs from the group keys coded from 3 to 9 ($GK_{3}^{q}$ to $GK_{9}^{q}$).

Figure 3b is a similar distribution diagram of Figure 2b as that of Figure 3a as it presents the results from the group key to the reader on the key tree; however, Figure 3b differs from Figure 3a in that the reader had a reading capacity of *r* = 4. In this example, the starting group key $GK_{3}^{q}$ of an incomplete subtree with the height of 1 can read five tags, which was larger than the number of tags that can be simultaneously read by a reader. Therefore, the group key $GK_{3}^{q}\;$was replaced by the subgroup keys $GK_{10}^{q}$ and $GK_{11}^{q}$ and the reader with the reading capacity of four tags generated grouping tags using group keys ranging from $GK_{4}^{q}$ to $GK_{11}^{q}$.

Subsequently, the following two strategies were employed to distribute $R_{e}-R_{f}+1$ number of group keys to the reader with a reading capacity of *r*.

- Only one group key was distributed to each reader used by a recipient to maximize the benefit of concurrent reading; consequently, a total of $\lceil \frac{R_{e}-R_{f}}{r-1}\rceil +R_{e}-R_{f}$ readers was required to generate the grouping proofs.
- Distribute one or multiple group keys to the reader to satisfy the condition that within all the readers, the total number of tags that can be encrypted by the key was less than the number of *r* tags, and only a minimal number of readers for recipients was required [41,42,43]; therefore, grouping proofs were generated using the least resources.

**Figure 4:**
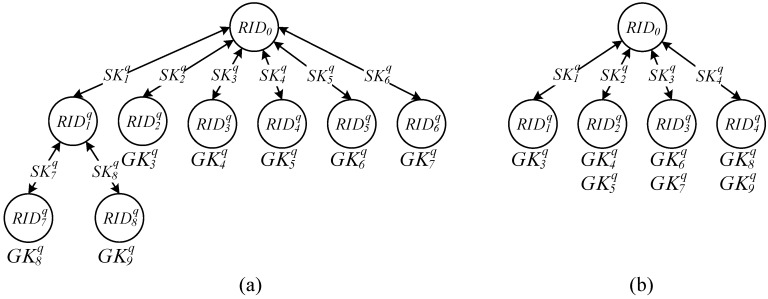
Read Tree with the reading capacity of six tags: (**a**) A single group key; (**b**) several group keys.

Finally, when the number of recipients’ readers was larger than *r*, reader $RID_{0}^{\;}$ cannot simultaneously transmit messages to all recipients’ readers. The method as indicated in Figure 2a in Section 2.1 was thus employed to form a read tree and code reader to solve the problem in which several readers simultaneously generate grouping proofs. To ensure the security of group key transmission and message transmission among readers, any reader $RID_{j}^{q}$ can use the key shared with the verifier to generate a session key $SK_{j}^{q}$ for encrypting messages transmitted between the parent node as well as to generate a maximum number of *r* keys ($\text{ranging from }SK_{j*r+1}^{q}$ to $SK_{j*r+r}^{q}$) for child node communication, and for encrypting messages transmitted between two readers. For example, Figure 4 presents the read tree in which each or several of the seven group keys $GK_{3}^{q}$, $GK_{4}^{q}$, $GK_{5}^{q}$, $GK_{6}^{q}$, $GK_{7}^{q}$, $GK_{8}^{q}$, and $GK_{9}^{q}$, as indicated in Figure 3a, were distributed to each reader. Figure 4a indicates that when the seven keys were processed into seven readers to enable all readers to concurrently read all the tags, transporter’s reader $RID_{0}^{\;}$ with a reading capacity for only six tags could not simultaneously transmit messages to seven readers owned by the recipients; instead, a middle reader was required for transferring messages. Thus, eight ($\lceil \frac{R_{e}-R_{f}}{6-1}\rceil +R_{e}-R_{f}=8$) readers were required in total. Pieces of proof were generated by $RID_{2}^{q}$ to $RID_{8}^{q}$; among them, reader $RID_{7}^{q}$ generated pieces of proof with tag codes $TID_{13}^{q}$, $TID_{14}^{q}$, and $TID_{15}^{q}$ from the distributed group key $GK_{8}^{q}$, with the proofs encrypted by session key $SK_{7}^{q}$ and transmitted back to the reader for decryption by parent node $RID_{1}^{q}$. Moreover, Figure 4b presents the results when several keys were written into the same reader; thus, only four readers ($RID_{1}^{q}$ to $RID_{4}^{q}$) were needed to simultaneously generate the pieces of proof for all tags. However, because a single reader such as $RID_{4}^{q}$ distributed two group keys $GK_{8}^{q}$ and $GK_{9}^{q}$, thus six pieces of proof for tags coded from $TID_{13}^{q}$ to $TID_{18}^{q}$ must be generated.

**Figure 5:**
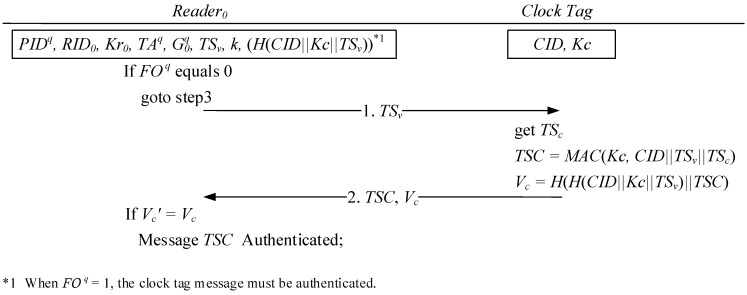
Signed timestamp acquired from trustworthy clock tag.

**Figure 6:**
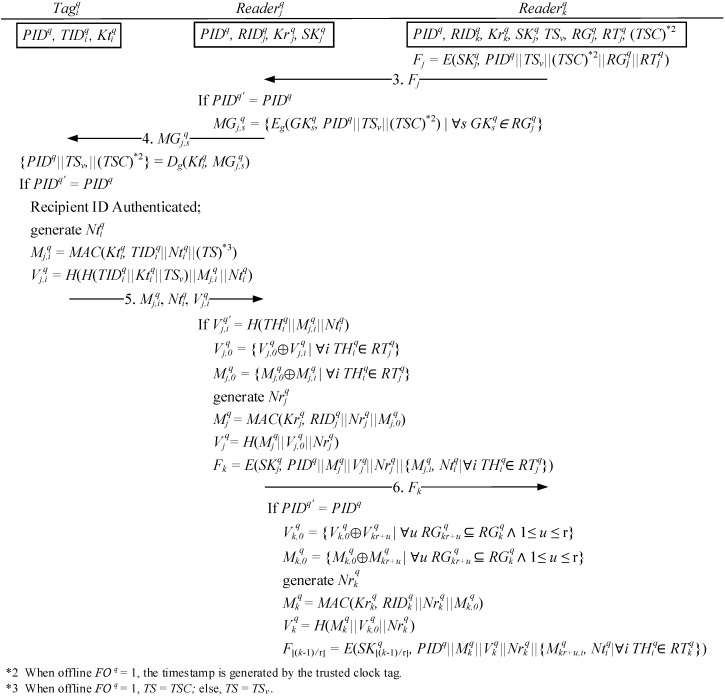
Generating grouping proofs by multilayered reader.

**Figure 7:**
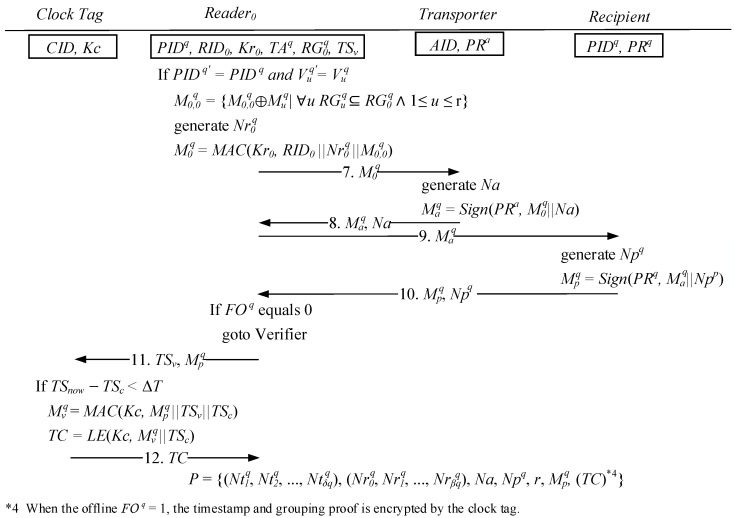
Affirm the tags and proofs signed by both sides and verify the time constraint.

After group keys were distributed, the following explains the three stages regarding grouping proof protocol OMRGP proposed in this study: how can $RID_{0}^{\;}$ generates a grouping proof under offline conditions in which the reader cannot be instantly connected to the backend verification server. From Figure 5, Figure 6 and Figure 7, the contents in the boxes at the top indicate that the contents were information already written into protocol during the initial setting and before the execution of the protocol. First, through third-party active clock tag, Stage 1 obtained the trusted start timestamp for the system. Second, Stage 2 gradually generated pieces of grouping proof and inspection pieces from the read tree’s leaf nodes, with the parent node combining all pieces of proof from child nodes until the tree root. Finally, Stage 3 incorporated affirming whether the grouping proofs signed by both the transporter and recipient and sent to the clock tag-grouping proof in the beginning was completed in time.

As illustrated in Figure 5, Stage 1 indicates that when transporter’s reader $RID_{0}^{\;}$ cannot connect to the verification server, the timestamp $TS_{v}^{\;}\;$written into the reader in the initial setting must be first transmitted to trusted clock tag to acquire the trustworthy initial time. After the clock tag receives the $TS_{v}^{\;}$ transmitted by the reader $RID_{0}^{\;}$, it uses the identification code *CID*, verifier’s shared key $K_{C}^{\;}$, tag’s current timestamp $TS_{c\;}^{\;}$, and the received timestamp $TS_{v}^{\;}$ to compute the signed timestamp *TSC*. The message verification code $V_{c}^{\;}=H(H\left(CID\left|\left|K_{c}^{\;}\right|\right|TS_{v}^{\;}\right)||TSC)$ is also computed and transmitted to the reader $RID_{0}^{\;}$ along with *TSC*. Finally, to authenticate the source of the received message *TSC*, the reader $RID_{0}^{\;}$ uses the clock tag check code $H\left(CID\left|\left|K_{c}^{\;}\right|\right|TS_{v}^{\;}\right)$ received from the verifier and the acquired *TSC* to compute the message verification code $V_{c}^{\;}$. 

At Stage 2, the reader $RID_{0}^{\;}$ uses the keys in the layered read-tree and child node (Figure 4) to encrypt recipient’s identification code $PID_{\;}^{q}$, timestamp $TS_{v}^{\;}$, signed timestamp TSC, group key set $RG_{j}^{q}$ for the child node reader $RID_{j}^{q}$, and tag verification code set $RT_{j}^{q}$ and transmits them to all child nodes until all leaf nodes have been reached. For example, as indicated in Figure 4a, the reader $RID_{0}^{\;}$ first uses the key $SK_{1}^{q}$ to encrypt $PID_{\;}^{q}$, $TS_{v}^{\;}$, TSC, $RT_{1}^{q}=\left\{TH_{13}^{q},TH_{14}^{q},TH_{15}^{q},TH_{16}^{q},TH_{17}^{q},TH_{18}^{q}\right\}$, and $RG_{1}^{q}=\left\{GK_{8}^{q},GK_{9}^{q}\right\}$, and then transmits them to the reader $RID_{1}^{q}$, which employs the session key $SK_{1}^{q}$ to decrypt the message and encrypt $PID_{\;}^{q}$, $TS_{v}^{\;}$, TSC, $RT_{7}^{q}=\left\{TH_{13}^{q},TH_{14}^{q},TH_{15}^{q}\right\}$, $RG_{7}^{q}=\left\{GK_{8}^{q}\right\}$, $PID_{\;}^{q}$, $TS_{v}^{\;}$, TSC, $RT_{8}^{q}=\left\{TH_{16}^{q},TH_{17}^{q},TH_{18}^{q}\right\}$, and $RG_{8}^{q}=\left\{GK_{9}^{q}\right\}$ by using the session keys $SK_{7}^{q}$ and $SK_{8}^{q}$, and then sending them to the leaf node readers $RID_{7}^{q}$ and $RID_{8}^{q}$. Subsequently, all leaf node readers collect the pieces of proof from the corresponding tags to generate a grouping proof, which is then transmitted to the upper levels and transmitted back to the reader $RID_{0}^{\;}$.

Since the activities of any two readers in the read-tree are identical, the algorithm uses any leaf node reader $RID_{j}^{q}$ in the read-tree receiving a request from the parent node reader $RID_{k}^{q}$ to generate a grouping proof, as shown in the steps in Figure 6. After the leaf node reader $RID_{j}^{q}$ uses its session key $SK_{j\;}^{q}$ to decrypt a message $F_{j}^{\;}$ transmitted from the reader $RID_{k}^{q}$ of the recipient $PID_{\;}^{q}$, all group keys in $RG_{j}^{q}$ are extracted and the multicast message $MG_{j,s}^{q}$ is encrypted using the keys $PID_{\;}^{q}$, $TS_{v}$, and TSC and transmitted to each tag to generate the pieces of proof.

When any tag $TID_{i}^{q}$ receives a multicast message $MG_{j,s}^{q}$ that is decrypted using the shared key $Kt_{i}^{q}$ of the verifier, the decrypted messages are checked to determine whether they contain the correct $PID_{\;}^{q}$ in order to verify the read message. When the message is successfully verified, the shared key $Kt_{i}^{q}$ of the verifier is employed to compute the pieces of proof $M_{j,i}^{q}$ assigned to a tag $TID_{i}^{q}$ generated by $RID_{j}^{q}$ for confirming the personal tag identification code $TID_{i}^{q}$, a randomly generated number $Nt_{i}^{q}$, and a timestamp (when the offline stamp and online stamp are TSC and $TS_{v}$, respectively). Subsequently, the hash value $H\left(TID_{i}^{q}\left|\left|Kt_{i}^{q}\right|\right|TS_{v}\right)$ for $TID_{i}^{q}$, $Kt_{i}^{q}$, and $TS_{v}$ are computed together with the pieces of proof $M_{j,i}^{q}\;$and a random number $Nt_{j,i}^{q}$ to generate the message verification code $V_{j,i}^{q}$ for the reader to reconfirm.

When the leaf node reader $RID_{j}^{q}$ receives response messages from the tags, the obtained $Nt_{i}^{q}$, $M_{j,i}^{q}$, and tag verification value $TH_{i}^{q}=H\left(TID_{i}^{q}\left|\left|Kt_{i}^{q}\right|\right|TS_{v}\right)$ transmitted from $RID_{k}^{q}$ (as indicated in Step 3) are computed to obtain $V_{j,i}^{q}$; in addition, the message verification code $V_{j,i}^{q^{\prime }}$ transmitted by the tags are, this time, employed to inspect the message integrity and verify which tags transmitted the messages, which is derived from the other group members, in order to prevent malicious users from exploiting any of the recipients’ tags that are not associated with this delivery, thereby blocking transmission of the proof. Subsequently, the reader $RID_{j}^{q}$ combines all of the pieces of proof $M_{j,i}^{q}$ and the verification code $V_{j,i}^{q}$ by incorporating the XOR operation of commutative law, both of which are generated by the group member tags, into pieces of proof $M_{j,0}^{q}$ without sequence and message verification code $V_{j,0}^{q}$. Through the shared key $Kr_{j}^{q}$ of the verifier, the pieces of proof $M_{j}^{q}$ generated by the reader are computed using the reader identification code $RID_{j}^{q}$ and the randomly generated numbers $Nr_{j}^{q}$ and $M_{j,0}^{q}$; along with $V_{j,0}^{q}$ and $Nr_{j}^{q}$, the message verification code $V_{j}^{q}$ is generated for all of the tags. The session key $SK_{j}^{q}$ is used to encrypt $PID_{\;}^{q}$, $M_{j}^{q}$, $V_{j}^{q}$, $Nr_{j}^{q}$, $M_{j,i}^{q}$, and $Nt_{i}^{q}$ for all group member tags, which are transmitted back to parent node reader $RID_{k}^{q}$.

After the parent node reader $RID_{k}^{\;}$ receives the response message $F_{k}^{\;}$ transmitted by child node reader $RID_{j}^{q}$, the session key $SK_{j\;}^{q}$ is first used to encrypt the message to confirm that the message contains the same recipient $PID_{\;}^{q}$ in order to ensure the correctness of the message. Subsequently, using the same method as the leaf node reader, all of the received pieces of proof $M_{j}^{q}$ and message verification codes $V_{j}^{q}$ of the child node reader are used to generate the pieces of proof $\;M_{k}^{q}$ for the reader, and the message verification code $V_{k}^{q}$ is transmitted with message $F_{\left\lfloor \left(k-1\right)/r\right\rfloor }^{\;}\;$ to the reader at the upper level.

As shown in Figure 7, Stage 3 indicates that after the reader $RID_{0}^{\;}$ receives a message from the recipient $PID_{\;}^{q}$, the tag verification value $TA^{q}$ generated by the verification server, and the $Nt_{i}^{q}$, $M_{j,i}^{q}$, $Nr_{j}^{q}$, and $M_{j}^{q}$ transmitted by the child node reader are computed to confirm whether they match the message verification code $V_{j}^{q}$ to reconfirm the message integrity and verify the tag message. In addition, the shared key $Kr_{0}^{\;}$ of the verifier is employed to generate the grouping proof $M_{0}^{q}$, which is then transmitted to the transporter’s tags depending on the results obtained from the identification code $RID_{0}^{\;}\;$of the reader, and a randomly generated number $Nr_{0}^{\;}$ and all of the excluded pieces of proof sent back from the child node.

When the transporter’s tag receives the grouping proof $M_{0}^{q}$ from the reader $RID_{0}^{\;}$, a random number Na is generated, and the transporter’s private key $PR^{a}$ is used with the signing function to compute $M_{0}^{q}$ and the signed proof $M_{a}^{q}$; subsequently, $M_{a}^{q}$ and Na are transmitted back to the reader $RID_{0}^{\;}$. After the reader $RID_{0}^{\;}$ receives the message $M_{a}^{q}$ signed by the transporter, $M_{a}^{q}$ is then transmitted to the recipient’s tag for signing. Using the randomly generated numbers $Np^{q}$ and $M_{a}^{q}$ and the private key $PR_{\;}^{q}$, the recipient computes the signed proof $M_{P}^{q}$; finally, $M_{p}^{q}$ and $Np^{q}$ are then transmitted back to the reader $RID_{0}$.

If the reader $RID_{0}$ cannot connect to the verifier when the receipt proofs and pick proofs are signed by the transporter and recipient, then the timestamp $TS_{v}$ and signed proof $M_{p}^{q}$ must be transmitted back to trusted clock tag to verify that parts of the cargo could not have been moved to other locations while the proofs were being generated. When the clock tag receives a message indicating that the difference between the system time and the time when grouping proof was initialized ($TS_{c}^{\;}$) is below the threshold value, the shared key Kc of the verifier can be employed to generate the final grouping proof $M_{v}^{q}$ for $M_{p}^{q}$, $TS_{v}^{\;}$, $TS_{c}^{\;}$, thereby providing evidence that all of the tags, the transporter, and the recipient are in the same interval. Using a light symmetric key encryption method, the key Kc encrypts the grouping proofs $M_{v}^{q}$ and $TS_{c}^{\;}$ into the message TC, which is transmitted back to the mobile reader and then to the verifier once a connection becomes available, for the protocol to be finalized. However, if the reader $RID_{0}$ can connect to the verifier when it receives the grouping proofs signed by the transporter and the recipient, then the clock tag is not needed and the grouping proof can be directly transmitted to the verifier to confirm whether the grouping proof has been completed within the time threshold.

### 2.3. Dispute Resolution Phase

When the transporter transmits the final grouping proof P to the verifier, the verifier confirms the integrity of the transporter, recipient, and cargo. First, when $FO_{\;}^{q}=0$, the shared key Kc of the clock tag is employed to decrypt in order to obtain the grouping proof $M_{v}^{q}$ and start time $TS_{c}^{\;}$ in message TC; subsequently, the recipient’s random number $Np_{\;}^{q}$ and public key $PK_{\;}^{q}$ are used to decrypt the grouping proof $M_{v}^{q}$ in order to obtain $M_{a}^{q}$, and the transporter’s random number Na and public key $PK^{a}$ are employed to decrypt $M_{a}^{q}$ in order to obtain the grouping proof $M_{0}^{q}$. According to the total number of tags δq, segments *k*, readers βq, and the maximum number of concurrent scans *r*, the read-tree and grouping key are surmised from and adopted for generating the proofs. In accordance with the proposed protocol, $M_{0}^{q}$ is recomputed and compared with the transmitted grouping proof $M_{0}^{q\prime }$ to determine whether the two are identical. Finally, according to the shared key Kc, start time $TS_{v}^{\;}$, and timestamp $TS_{c}^{\;}$ of the clock tag, the message verification code $M_{v}^{q}=MAC\left(K_{c}^{\;},M_{p}^{q}\left|\left|TS_{v}^{\;}\right|\right|TS_{c}^{\;}\right)$ is computed to reconfirm whether the code corresponds with the authentication message provided by the clock tag, thus effectively completing the grouping proof. By contrast, when $FO_{\;}^{q}=0$, the verifier first computes whether the time difference between the current system time and timestamp $TS_{v}^{\;}$ is below the threshold value; subsequently, $M_{0}^{q}$ is computed by the read-tree, and $M_{v}^{q}$ is confirmed to complete the reinspection through the proofs signed by both parties and the computed random number $M_{0}^{q\prime }$.

Therefore, in the process of generating the grouping proofs, the transporter and recipient each verify all of the involved tags and use their personal private keys to sign the proofs; thus, the grouping proofs guarantee the rights of both parties. Specifically, when cargo is received by the recipient, the transporter has undeniable proof that the recipient has received the cargo. In addition, if the recipient needs to return cargo through the transporter, the same protocol can be applied, except that the roles of recipient and transporter are swapped. The recipient also has the signed proof indicating that the cargo has been returned to the transporter, thus preventing the transporter from denying that cargo has been retrieved.

## 3. Security and Performance Analysis

The proposed method and the Internet connection method for the verifier use secure frameworks and can therefore be trusted. Extant mechanisms can also be employed so that the transporter’s reader $RID_{0}^{\;}$ can extract the recipient’s identification code $PID_{\;}^{q}$, timestamp $TS_{v}^{\;}$, tag check code $TA_{\;}^{q}$, and group of keys $G_{0}^{q}$ by using the current security verification procedure to ensure that the connection is secure before the protocol proceeds. The following analysis is primarily focused on determining whether the protocol proposed in this study can prevent most known malicious attacks aimed at exploiting grouping proofs transmitted between tags and readers, and whether it can guarantee anonymity and message integrity.

Prevent Replay Attack

Malicious users intercept a message containing a previously generated grouping proof through an eavesdropping, and the previously captured message is replayed to produce grouping proofs for nonexistent tags. However, because any piece of proof for the tags incorporates a random number $Nt_{i}^{q}$, timestamp TSC, or $TS_{v}$ generated by tags, the reader can thus detect errors and ignore the replay message by using the message check code transmitted from the verifier to authenticate the received message.

Prevent Tag Impersonation

The tags generate the pieces of proofs $MAC\left(Kt_{\;i}^{q},TID_{\;i}^{q}\left|\left|Nt_{\;i}^{q}\right|\right|TS\right)$ and a message verification code $V_{j,i}^{q}$ from the shared key $Kt_{i}^{q}$ of the verifier, and the reader stores the message verification code required for reading the tags, which is generated by the verifier. Because malicious users do not have the required shared key $Kt_{i}^{q}\;$of any tag in the tag group, the pieces of proofs generated by impersonated tags cannot pass the reader’s or the verifier’s authentication process.

Prevent Multi-session Attack

If multiple readers simultaneously generate grouping proofs, the leaf node reader stores the tag check codes of all of the members in this tag group; thus, tags that are not assigned to the group cannot pass the authentication process; and thus, tag impersonation attacks are ineffective. Consequently, malicious users cannot forge extra grouping proofs by crisscrossing pieces of proofs derived from two different groups.

Prevent Denial of Proof

In addition to generating pieces of proof for every tag, the protocol also generates the message verification code $VA_{j,i}^{q}$ with the tag verification code $H\left(TID_{i}^{q}\left|\left|Kt_{i}^{q}\right|\right|TS_{v}\right)$. Although the leaf node reader has no shared key $Kt_{i}^{q}$ for the tags and cannot generate tag verification codes, using the cargo integrity check code $\;TH_{i}^{q}=H\left(TID_{i}^{q}\left|\left|Kt_{i}^{q}\right|\right|TS_{v}^{\;}\right)$ provided by the verifier, it can confirm whether a response message has cargo tag members that do not belong to this delivery, but are instead generated by a malicious user. Therefore, the condition of authentication failure being generated by the verifier despite the existence of all legitimate tags is prevented.

Prevent Concurrency Attack

When two readers simultaneously use the same tags, parameter confusion can occur, which enables a malicious reader to scan tags by crisscrossing tags, and block grouping proofs. However, in the proposed protocol, no cargo tag has a temporary parameter, and the reader needs to communicate with the tags only once to generate the pieces of proof. Therefore, it is impossible for a concurrency attack to occur.

Anonymity

In the proposed protocol, all messages used by the reader are multicast messages that do not contain specific tag information. In addition, the pieces of proof $M_{i}^{q}$ and message check code $V_{j,i}^{q}$ transmitted by any tag $TID_{i}^{q}$ are computed from using hash functions, along with the random numbers $Nt_{i}^{q}$ generated each time and a shared key $Kt_{i}^{q}$; thus, the anonymity of the cargo tags can be protected. In the final signed message, the confidentiality of the transporter’s and recipient’s tags is protected by random numbers Na and $Np_{\;}^{q}$, respectively.

Prevent Tracking Attack

The protocol proposed in this study can protect the anonymity of all the involved tags. The messages transmitted by a tag change according to the random number $Nt_{i}^{q}$, which is generated each time a message is sent, and the reader also uses a different session key for every message. Therefore, the relationship among the messages containing proofs for any tag cannot be obtained by analyzing multiple grouping proofs; thus, the protocol ensures the confidentiality of the location of the cargo tags to prevent the cargo from being tracked by malicious users.

Message Integrity

The pieces of proof $M_{j,i}^{q}$ transmitted back to the reader by the tags are require a random number $Nt_{i}^{q}$ in order to be calculated; thus, when a malicious user intercepts the random numbers, despite the pieces of proof being generated by legitimate tags, the proofs cannot be successfully reconstructed by the verifier because the random numbers are different for every message. Therefore, the message verification code $V_{j,i}^{q}$ is employed to ensure that a response message has not been modified in order to ensure the message integrity.

Table 2 shows the OMRGP proposed in this study and other grouping proof methods to compare whether they can protect against the major types of attacks targeting grouping proofs: replay attack, tag impersonation attack, multisession attack, denial of proof, concurrency attack, and tracing attack. The protocols can protect against those marked with an “O”; those marked with an “X” are a security threat; and those marked with “∆” can be are not a threat so long as certain conditions are satisfied.

**Table 2:** Comparison table indicating the security of grouping proof.

Protocol	Replay Attack	Tag Impersonation	Multi-Session Attack	Concurrency Attack	Denial of Proof
Burmester *et al.* [10]	O	O	O	X	∆^2^
Saito *et al.* [13]	X	X	O	X	X
Lin *et al.* [18]	O	O	O	O	X
Sun *et al.* [19]	O	O	O	∆^1^	X
Hermans *et al.* [20]	O	O	O	X	X
Lo *et al.* [21]	O	O	O	O	X
Ma *et al.* [22]	O	O	O	O	X
Chien *et al.* [24]	O	O	O	X	X
Peris-Lopez *et al.* [26]	O	O	O	O	X
Piramuthu [27]	O	O	X	X	X
Sundaresan *et al.* [28]	O	O	O	O	O
Yen *et al.* [32]	O	O	O	O	∆^2^
Leng *et al.* [37]	O	O	O	X	X
Huang *et al.* [44]	O	X	O	X	X
OMRGP	O	O	O	O	O

Note: ∆^1^: Not overwriting the proofs from different readers; but the random numbers generated by proofs may still be overwritten; ∆^2^: Filters proofs that do not belong to a group of tags, but cannot prevent a denial of proof attack because of the compromised proof integrity.

Table 2 shows that the grouping proof proposed in this study can protect against all major attacks currently in use. In the method proposed by Saito *et al.* [13], the tags generate messages but do not use random numbers that change for every message; consequently, malicious users can generate counterfeit tags by replaying old messages to generate grouping proofs [18,27,31]. The method proposed by Huang and Ku [44] can be exploited by replacing parts of the pieces of proof to forge tags [26,45] and authentication can be avoided if the verifier has listed tag as redundant in its cyclic redundancy check. Peris-Lopez *et al.* [15] showed that the method proposed by Piramuthu [27] was flawed because it enables malicious users to eavesdrop and intercept pieces of grouping proofs by crisscrossing two identical time intervals to forge an additional third proof. In addition, according to the methods proposed by Saito *et al.* [13] and Piramuthu [27], tags are read and written multiple times to generate grouping proofs; this causes the problem in which the previously written content can be overwritten by other readers [21]. Moreover, various methods for generating grouping proofs [10,20,24,37,44] require the reader to read the tags more than twice when generating grouping proofs. However, the tags cannot verify readers; thus, when several readers simultaneously generate grouping proofs for the same tag, concurrency attacks can arise, which can prevent grouping proofs from being generated because the contents in previous tags are overwritten by subsequent readers. The method proposed by Sun *et al.* [19] requires the read group to be inspected every time tags generate proofs; thus, the proofs read by different readers are not overwritten; however, the random numbers are not subjected to the same security check in the previous step and may therefore be overwritten. Because the proposed method can complete the grouping proofs by reading and writing on the tags and because random numbers are also used in addition to the identification codes used in the prior authentication process, the proposed protocol prevents erroneous grouping proofs from being generated, thereby protecting against replay attacks, tag impersonation, multisession attacks, and concurrency attacks.

In addition to the correctness of the generated proofs, the readers do not authenticate the response message tags in the grouping proof methods of [13,18,19,20,21,22,24,26,27,37,44]; thus, if response messages generated from tags that do not belong to the specific group of tags are included, the verifier rejects the messages and discards the proofs, resulting in a denial of proof [28]. Yen *et al.* [32] and Burmester *et al.* [10] have proposed that message integrity must be authenticated to prevent parts of a message from being modified, which causes the problem in which legitimate proofs cannot be authenticated by the verifier, resulting in a denial of proof [28]. Therefore, in the present study, readers were employed to verify all of the collected tags to prevent including tags that do not belong to the group and to avoid denial of proof from occurring. Finally, the method proposed by Sundaresan *et al.* [28] can protect known attacks on grouping proofs. However, as shown in Table 3, because the proposed method cannot enable all of the involved proof tags to reduce the time for generating grouping proofs through parallel computing, therefore, attackers can exploit this time difference to generate legitimate grouping proofs from a group of tags that do not exist in the same time and location [36]; consequently, this method is inapplicable to SCM where large volumes of cargo are involved.

**Table 3:** Comparison of grouping proof performance.

Protocol	Anonymity	Tracking Attack	Offline	Order Independent	Simultaneity
Burmester *et al.* [10]	O	O	O	X	∆^4^
Saito *et al.* [13]	O	∆^3^	X	X	X
Lin *et al.* [18]	X	X	O	X	X
Sun *et al.* [19]	O	O	O	O	O
Hermans *et al.* [20]	O	O	O	O	O
Lo *et al.* [21]	O	O	O	X	X
Ma *et al.* [22]	O	O	O	X	X
Chien *et al.* [24]	O	∆^3^	X	X	X
Peris-Lopez *et al*. [26]	O	O	X	X	X
Piramuthu [27]	X	X	X	X	X
Sundaresan *et al*. [28]	O	O	O	X	X
Yen *et al.* [32]	O	O	X	O	O
Leng *et al.* [37]	X	X	X	O	X
Huang *et al.* [44]	X	X	O	X	X
OMRGP	O	O	O	O	O

Note: ∆^3^: Single message that features anonymity; however, relevance among tags with messages from different sessions can be used to track tag movement; ∆^4^: Only parts of the tags in the group can concurrently compute pieces of proof.

Table 3 indicates that the proposed system can prevent cargo from being tracked and provide anonymity while operating in the offline phase; simultaneously, pieces of proof that do not need to follow the tag sequence can be generated to achieve the five types of security characteristics involved in processing grouping proofs. However, to ensure confidentiality, the system must be designed to prevent malicious attackers from obtaining the identity of the tag owner by eavesdropping on grouping proofs. Because many grouping proof methods [18,27,37,44] involve directly transmitting identification codes to the readers without first anonymizing the tag owner’s identity; consequently, the cargo owners’ private information can be stolen and the location of their cargo can be tracked [28]. Moreover, although the methods proposed by Chien *et al.* [24] and Saito *et al.* [13] ensure tag anonymity and thus ensure that malicious attackers cannot track the tagged cargo simply by monitoring the identification codes of the tags, nevertheless, because the request acknowledgement response messages sent by the tags are identical, malicious attackers can track the location of the tagged cargo by eavesdropping on multiple messages [26]. Therefore, this study incorporated random numbers into the messages to scramble the responses for preventing from being tracked in the supply chain, thereby achieving location privacy.

According to the method proposed by Peris-Lopez *et al.* [26], a reader must be able to connect to the verifier for it to obtain a timestamp of when the proofs were generated; subsequently, the grouping proofs are immediately sent to the verifier to compare the time [22]. Similar grouping proof methods [13,24,27,32,37] also require immediate authentication from the verifier, and are unsuitable for generating grouping proofs in the offline phase.

The grouping proofs proposed by Saito *et al.* [13] and Piramuthu *et al.* [27] pertain to conventional proofs generated by all tags on site one after another; thus, the verifier must verify the tags in the order that they were generated [29]; furthermore, methods for generating grouping proofs [10,18,21,22,24,26,44] typically have a particular sequence. Because the method proposed by Leng *et al.* [37] involves unicasting messages to tags, the participating members cannot concurrently conduct computation [19]. In the method proposed by Burmester *et al.* [10], the tree structure can permit only a few tags in the group to concurrently compute the pieces of proof, and the reader must follow the predetermined sequence when collecting the proofs. Therefore, the present study adopted the multicasting method to simultaneously generate the pieces of proof for all tags and can collect the grouping proofs without adhering to any sequence through the XOR operation of commutative law.

## 4. Effectiveness Analysis

Because the grouping proofs generated in sequence require the time complexity O(*m*!) when being authenticated by the verifier, this section compares the proposed OMRGP method only with those grouping proof methods that do not require a predetermined sequence [19,20,32,37] to examine the computing and transmission time for the tag and reader to generate proofs. To ensure that the comparison is objective, the experiments were conducted under the following constraints: each method involved using a reader that can scan *r* tags [39] to generate grouping proofs for *m* tags [46] at a rate of 3.55 M clock cycles per second. In addition, an error-correcting code and asymmetric encryption function with the same security strength (2^80^ bits) were employed.

Specifically, *T_SE_* denotes the computation time for conducting symmetric encryption and decryption [47], *T_EC_* indicates the time for conducting elliptic curve encryption and decryption [48], *T_G_* represents the time for encrypting and decrypting a group key [49], *T_RNG_* denotes the required time for generating a random number [50], *T_H_* is the computation time for executing a hash function [47], and *T_SIG_* indicates the required time for proof signing [51]. In addition, because XOR logic operation can be neglected compared to the aforementioned computation time, the formulas in Table 4 do not consider the required time for this type of operand. To simplify the comparison, the computing capacity of the reader was adopted to present the required computation time for devices with a powerful arithmetic capability, as demonstrated by the additional timestamps used in the various methods.

**Table 4:** Computational capacity of grouping proof tags (with *m* number of tags).

Name of the Method	Cargo Tag	Mobile Reader
Sun *et al.* [19]	$\lceil m/r\rceil \left(2T_{SE}+T_{RNG}\right)$	$T_{SE}+2T_{H}$
Hermans *et al.* [20]	$\lceil m/r\rceil \left(2T_{EC}+2T_{RNG}\right)$	$T_{SIG}+T_{RNG}$
Yen *et al.* [32]	$\lceil m/r\rceil \left(7T_{RNG}\right)$	$2T_{SIG}+\text{m}\left(T_{RNG}\right)+5T_{RNG}$
Leng *et al.* [37]	$m\left(2T_{H}+T_{RNG}\right)$	$m\left(T_{H}\right)+\frac{m\left(T_{H}\right)}{r}+T_{H}$
OMRGP	$T_{G}+3T_{H}+T_{RNG}$	$T_{G}+2T_{SIG}+3T_{SE}+7T_{H}+3T_{RNG}$ $\left(\lceil \log_{r}\left(m/r\right)\rceil \right)\left(2T_{SE}+2T_{H}+T_{RNG}\right)$

**Figure 8:**
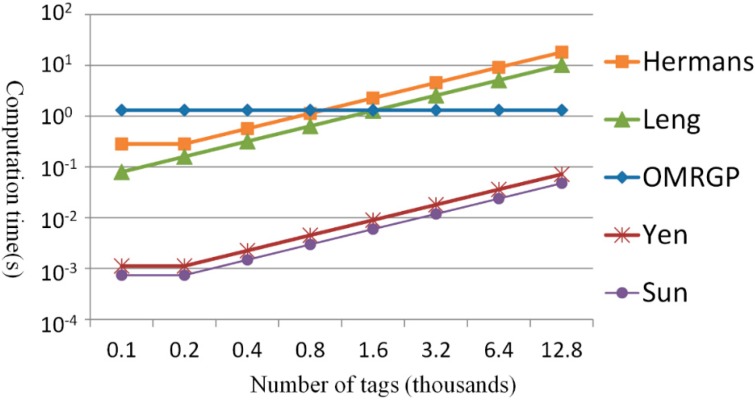
Comparing the computational load of the tags.

**Figure 9:**
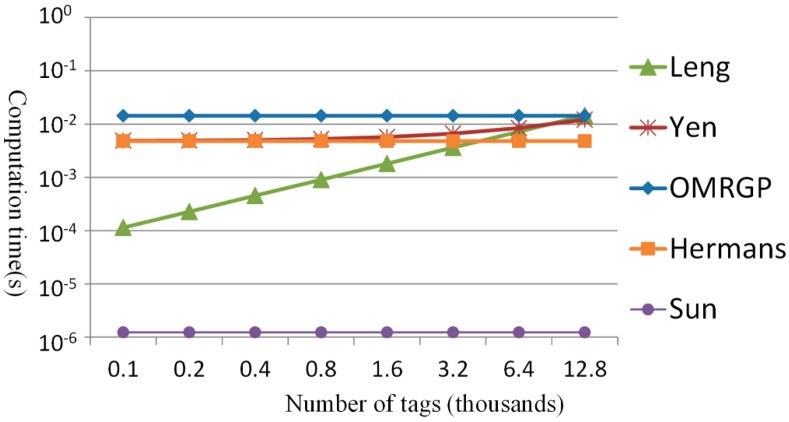
Comparing the computational load of readers.

Table 4 indicates the computational capacity of *m* tags according to the grouping proofs generated by the reader with *r* capacity for the maximum number of tags that can be scanned concurrently. Therefore, for the OMRGP method proposed in this study, each reader can manage a maximum of *r* and thus only one multicast is to be broadcasted to all tags. The grouping proof methods in [19,20,32] also send multicast messages to all tags; however, when *m* > *r*, the reader must transmit the message multiple times; thus, *m* tags required a computation time of ⌈m/r⌉ times. According to the method proposed by Leng *et al.* [37], a reader must send different messages to each tag, and each tag requires its own computation, in that *m* tags ultimately require *m* times of computation time. By contrast, Leng *et al.* indicated that the reader should assign messages to each tag; Yen *et al.* verified the identification code for individual tags, in which the computational capacity of the reader increased with the number of tags; and Hermans *et al.* and Sun *et al.* have employed methods in which identical messages were broadcast to all tags; thus, the required computational capacity for the reader to generate grouping proofs remained constant. The proposed method was designed for operation in a multilayered reader; despite a similar message is broadcast, the readers are required to communicate with other readers, thereby increasing the computational capacity to $\log_{r}m/r$ times. In Figure 8 and Figure 9, readers with a maximum reading capacity of 200 tags were employed to analyze the computing time required by various methods when the number of tags and group tags in the reader doubled from 100 each time until the quantity reached 12,800. Since we use a group key with the tree height of 2, a reader can multicast message to 200 tags within the capacity that can be read by the reader. In the following simulations, 100 tags and 200 tags need two readers, 400 tags need three readers, 800 tags need five readers, 1600 tags need nine readers, 3200 tags need 17 readers, 6400 tags need 33 readers, and 12,800 tags need 65 readers.

According to Figure 8, when the proposed OMRGP method involved fewer than 200 tags, more computing time was required because the group key has to be decrypted. When the number of tags exceeded 200, the number of grouping proofs [19,20,32,37] increased with the number of tags; thus, the computing time and tag number were linearly related. Therefore, when the grouping proofs were generated for an extreme number of tags, the tag computational capacity of the proposed OMRGP method was more efficient compared with the other methods. In addition, Figure 9 indicates the computational capacity of the reader in generating grouping proofs. Although the same message was broadcast, the required computation time was more than that of the other multicast methods [19,20,32] because the group messages must be encrypted, messages must be transmitted between readers, and proofs must be signed by both the transporter and the recipient. Because the method proposed by Leng *et al.* [37] adopted a unicast method, the required computation time under conditions involving extreme number of tags was higher than that required from using OMRGP method.

This study subsequently compared the required transmission time for the proposed OMRGP with that of the other methods, for which *L_ID_* denotes the length of a tag identification code (based on ISO-18000-6), *L_SE_* is the message length after applying symmetric encryption, *L_EC_* indicates the message length after applying elliptic curve encryption, *L_G_* represents the message length after performing group key encryption, *L_RNG_* indicates the message length for a random number, *L_H_* represents the message length of a hash function, and *L_SIG_* represents the required message length after signing the proof.

Table 5 indicates the transmission capacity of the grouping proofs generated by *m* tags. Because the proposed OMRGP method adopted a multilayered grouping proof structure, a maximum or *r* tags were distributed to each reader; thus, compared with the other methods, increasing the cargo volume did not increase the transmission time from the tags to the verifier. Moreover, in the transmission from the reader to tags, a read-tree was employed; consequently, the transmission time between readers increased $\lceil \log_{r}\left(m/r\right)\rceil$ times. In the methods proposed by Hermans *et al.*, Yen *et al.*, and Sun *et al.*, the readers could not manage *m* tags simultaneously; consequently transmissions were repeated ⌈m/r⌉ times. According to the subgrouping proof of Leng *et al.*, the reader transmitted the message ⌈m/r⌉+m times. For the sake of objectivity, all methods adopted the electronic product code Class-1 Generation 2 (EPC Class-1 Gen2), with the network bandwidth of the tags and readers set to 160 and 640 kbps, respectively [38]. The message lengths for *L_ID_*, *L_SE_*, *L_RNG_*, and *L_H_* were arbitrarily set at 64 bits, and the message lengths for *L_ECC_* and *L_G_* were arbitrarily set at 192 and 1024 bits, respectively.

**Table 5:** Transmission capacity of *m* grouping proof tags.

Name of the Method	From Tag to Reader	From Reader to Tag (or Reader)
Sun *et al.* [19]	$m\left(L_{ID}+2L_{SE}\right)$	$\lceil m/r\rceil \left(3L_{H}\right)$
Hermans *et al.* [20]	$m\left(2L_{EC}+L_{RNG}\right)$	$\lceil m/r\rceil \left(L_{RNG}\right)$
Yen *et al.* [32]	$m\left(4L_{RNG}\right)$	$\lceil m/r\rceil \left(3L_{RNG}\right)$
Leng *et al.* [37]	$m\left(2L_{ID}+L_{H}+L_{RNG}\right)$	$\lceil m/r\rceil \left(2L_{ID}+L_{RNG}\right)+m\left(L_{ID}+L_{H}\right)$
OMRGP	$r\left(2L_{H}+L_{RNG}\right)$	$L_{G}+\left(\lceil \log_{r}\left(m/r\right)\rceil \right)\left(2L_{SE}\right)$

**Figure 10:**
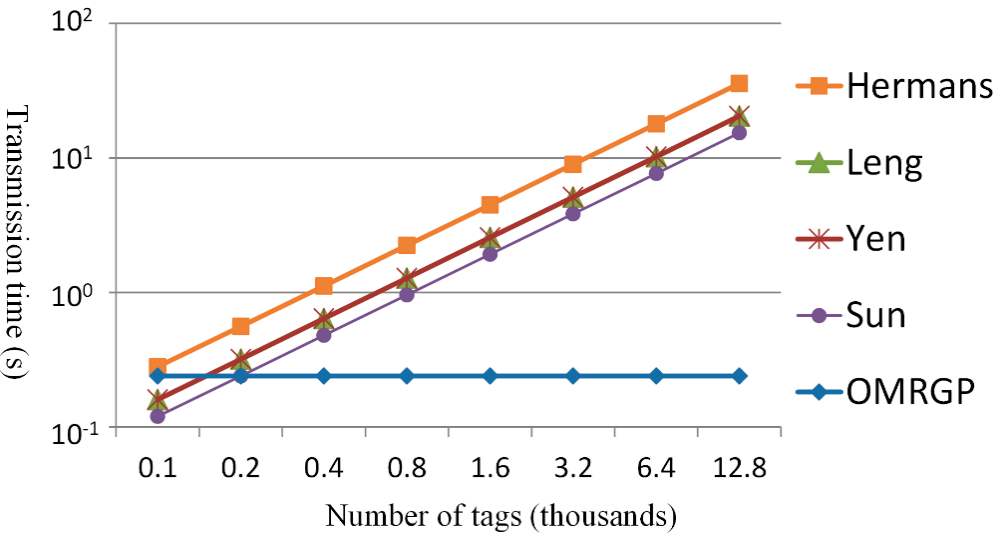
Comparing the message quantity of the collected tags.

**Figure 11:**
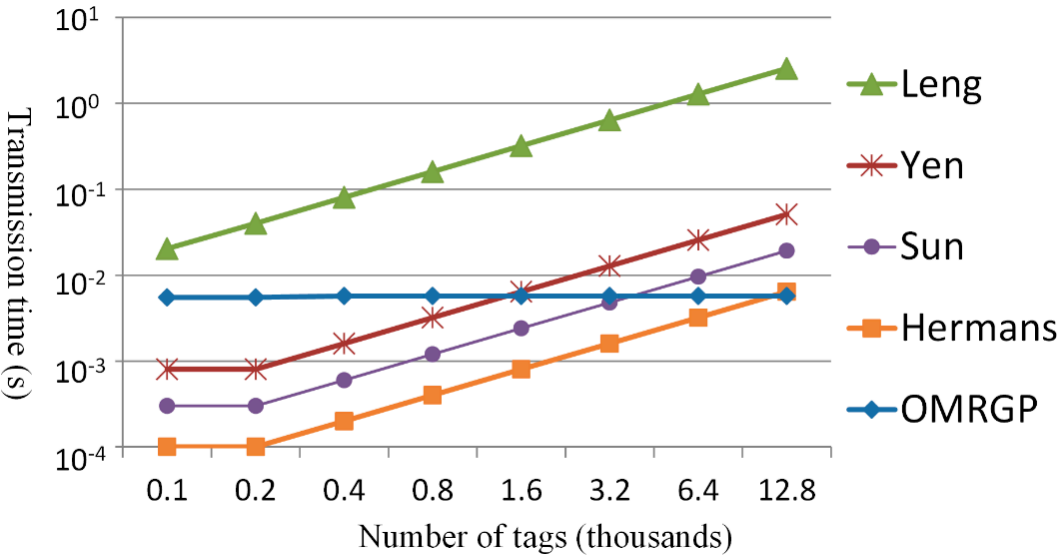
Comparing the message quantity transmitted by the reader.

Figure 10 and Figure 11 show the transmission time for the tags to generate the grouping proofs. The methods of Sun *et al.* and the one proposed in the present study transmitted messages that were identical in length (3×64=192 bits); however, the proposed method divided all of the tags into several groups and every group could process concurrently. When the number of tags exceeded 200, the time for the reader to collect the tags, according to Sun *et al.*, exceeded the fixed transmission time suggested in the proposed OMRGP method. In addition to the method proposed by Sun *et al.*, the other three methods had to transmit messages that were longer than 192 bits. Hence, when >200 tags were involved, the transmission time of the proposed method was shorter than that of the other methods. Figure 11 depicts the time required for the reader to transmit messages. Leng *et al.* [37] did not adopt a multicasting protocol for transmitting messages, which increased the reader’s transmission time with an increased number of tags. When more than 200 tags were involved, the grouping proof methods of [19,20,32] were not able to scan all tags in one shot because of the reading capacity of the readers, which divided and read for several cycles, thereby increasing the transmission time. In this present study, grouping proofs were generated using the group key and the multilayered read-tree. When there was only a few tags involved, the proposed OMRGP method took longer to read than that other multicast methods; however, an observation can be made that the OMRGP method is more efficient compared with the other methods when a considerable number of tags is involved.

**Figure 12:**
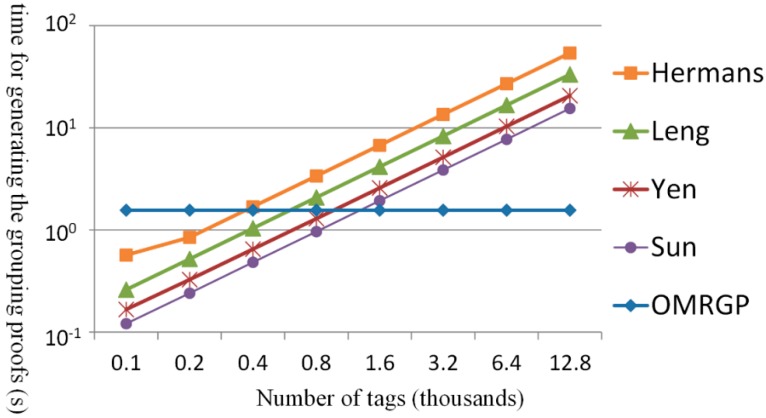
Comparing the time for generating grouping proofs.

Figure 12 shows the total amount of time consumed when several tags generated the grouping proofs, including the computing and transmission times of the tags and readers involved. In the OMRGP method, although an increased time was taken for computation, the transmission capacity was evidently larger than the computational capacity and thus prevented the time for generating grouping proof to increase considerably with an increased number of tags. In addition, the OMRGP method features a mechanism for verifying tags. According to Sun *et al.*, readers that were not required to verify tags reduced computation load; however, the time for generating grouping proofs when an extreme number of tags were involved exceeded the time for the OMRGP method. Yen *et al.* employed a random function to generate grouping proofs; groups could not be selected under their method; thus, under the condition in which a mechanism for verifying tags existed, the effectiveness of OMRGP method becomes more obvious once the number of tags exceeds a certain threshold. Leng *et al.* adopted a unicast method, in which the reader was required to perform a high number of transmissions; therefore, in less time than it would take unicast grouping proofs, the OMRGP method can generate grouping proof in advance and when there are fewer tags. Finally, Hermans *et al.* adopted a high elliptic curve encryption and decryption to generate grouping proofs, demonstrating the effectiveness of the OMRGP method when there are fewer tags.

## 5. Conclusions

This study proposed a method for generating multilayered grouping proofs to solve the disputes over the loss of cargo when high-quantity shipments are transferred in the supply chain. Through the layered parallel scans, the requirement in which the generated grouping proofs must be read in batches because of the maximum tag-reading capacity constraint on a reader in the supply chain environment can be solved. Group keys were employed to distribute the tags corresponding to each reader to ensure that the tags are not repeatedly read, thus exceeding the time threshold. In addition, both the transporter and recipient were allowed to verify the cargo and sign the proofs to guarantee the integrity of the grouping proof. The anonymity and message integrity characteristics of the OMRGP method can defend against most of the currently known attacks on grouping proofs: replay attack, multi-session attack, tag impersonation attack, denial of proof, and tracing attack. The OMRGP method overcame the problem of at least one type of characteristic not complying with the security standards, a problem possessed by most studies. This study also analyzed the computation load of the tag and reader. The effectiveness of grouping proof protocols were compared, and the results show that when an extreme number of tags are involved, the increase in the number of tags did not evidently increase the time for generating grouping proofs under the proposed protocol. Consequently, the protocol can be applied to SCM to reduce the time required to generate grouping proofs, and prevent exceeding the time threshold value for generating grouping proofs, thus preventing attackers from hijacking tags when the grouping proof is being processed, causing grouping proof problems.
